# Supplementary material for: Cooperative tumour cell membrane targeted phototherapy
Source: Nat Commun. 2017 Jun 19;8:15880. doi: 10.1038/ncomms15880 (PMC5481829; doi:10.1038/ncomms15880)
Supplement: Supplementary Information [file ncomms15880-s1.pdf]

Type of file: pdf
Title of file for HTML: Supplementary Information
Description: Supplementary Figures, Supplementary Methods and Supplementary References

Type of file: pdf
Title of file for HTML: Peer Review File
Description:

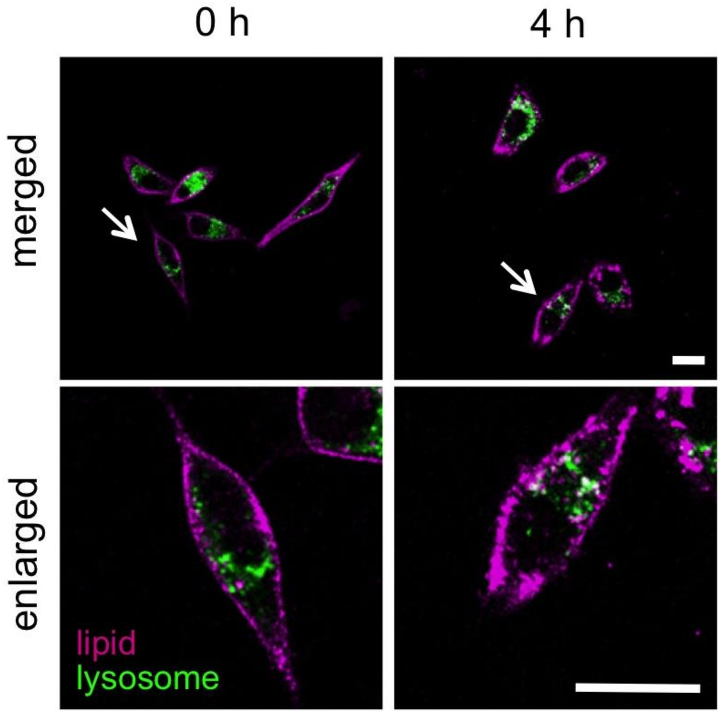

Supplementary Figure 1. Localization and retention of synthetic receptor-lipid conjugates (SR-lipids) delivered onto plasma membranes by fusogenic liposomes (FLs). Confocal fluorescent microscopic images of HeLa cells 0 h (immediately) and 4 h after FL-mediated delivery of fluorophore-lipids (magenta). Free liposomes were washed completely after treating the cells with fluorophore-FLs for 1 h. Bottom images showed the cells pointed by arrows in top images. Lysosomes were stained with LysoTracker (green). Scale bars represent 20 µm.

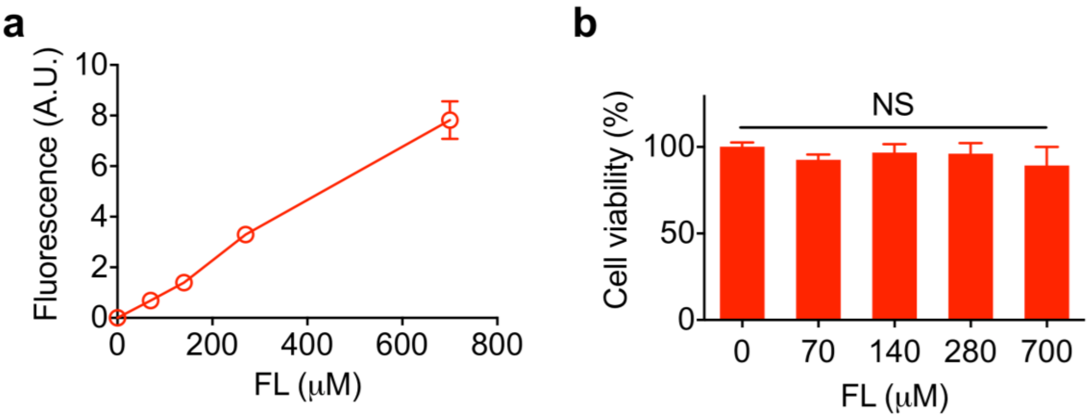

Supplementary Figure 2. Dose-dependent membrane localization and cellular viability of SR-lipids. (a) Fluorescence quantification of HeLa cells treated with fluorophore-SA after FL-mediated biotin-lipid delivery at different lipid concentrations. Data are means ± s.e.m. (n = 5). (b) Cell viability after FL-mediated delivery of biotin-lipids at different lipid concentration. The cell viability was evaluated using LIVE/DEAD cell assay. Data are means ± s.e.m. (n = 3; NS, not significant).

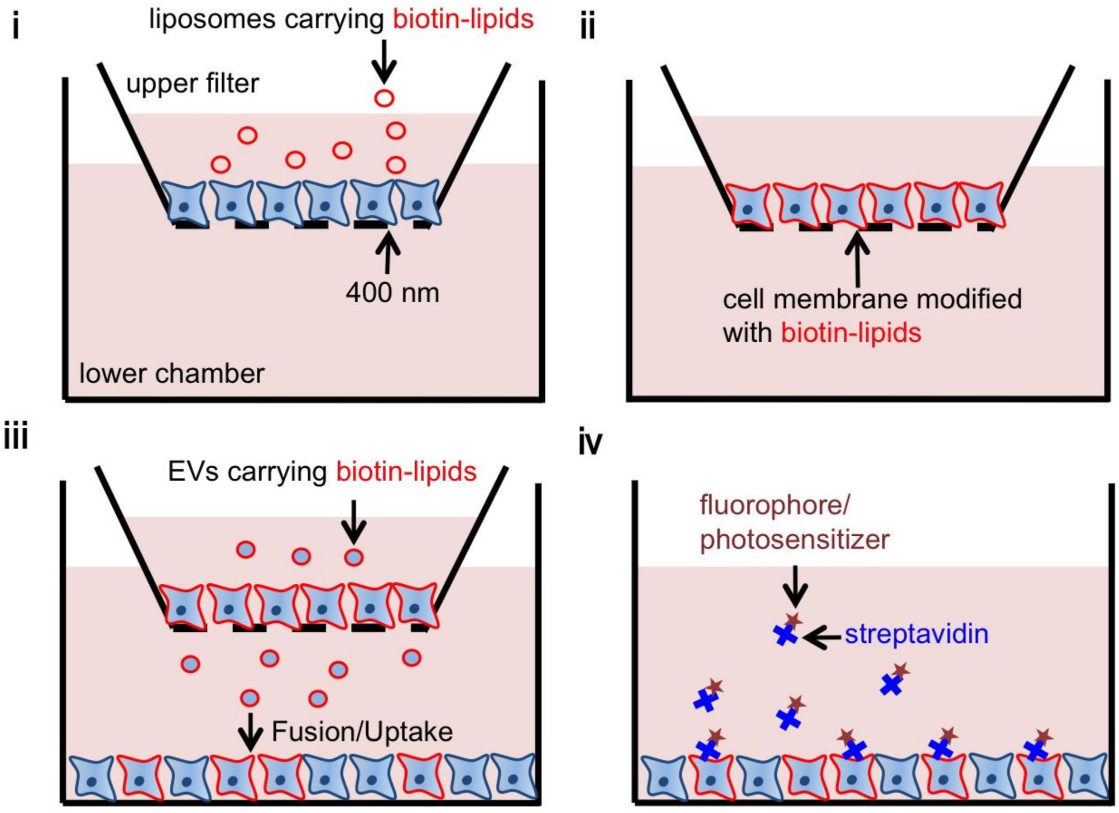

Supplementary Figure 3. Scheme of transwell experiments. Firstly, cells on the upper filter with 400-nm pores were treated with each type of liposomes incorporated with biotin-lipids (280 μM) for 1 h **(i)**, followed by through washing of free liposomes **(ii)**. The upper filter was then co-incubated with the lower chamber for 4 h to allow secretion of extracellular vesicles (EV) incorporated with biotin-lipids by cells on the upper filter and their transfer to cells in the lower chamber (iii). Then, the cells were treated with fluorophore(Alexa594)-SA (50 μM) for 1 h to identify the presence of biotin-lipids on the cell surface or photosensitizer(Ce6)-SA (50 μM) for 1 h to perform photodynamic therapy (PDT), respectively (iv).

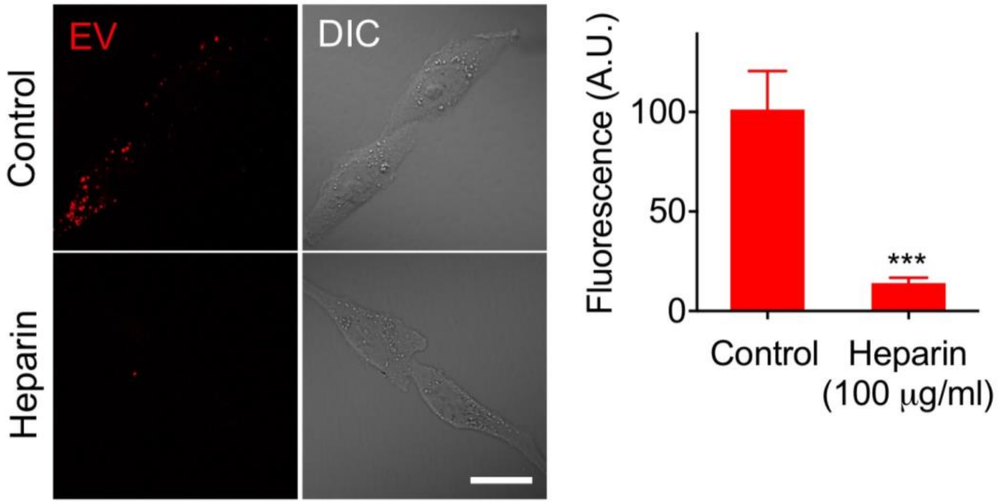

Supplementary Figure 4. Heparin treatment for inhibition of cellular uptake of EVs. Confocal fluorescent microscopic images and fluorescence quantification of HeLa cells treated with EVs containing fluorohpore-lipids in the presence of heparin. EVs from cells treated with FLs containing fluorohpore-lipids were isolated from cell culture medium using ultracentrifugation method. Fresh cells were treated with EVs containing fluorophore-lipids in the absence (control) or presence of 100 μg/ ml heparin for 2 h, and then imaged with confocal microscopy. Data are means ± s.e.m. (n = 20, ***p<0.001, Student's *t* test). Scale bar represents 20 μm.

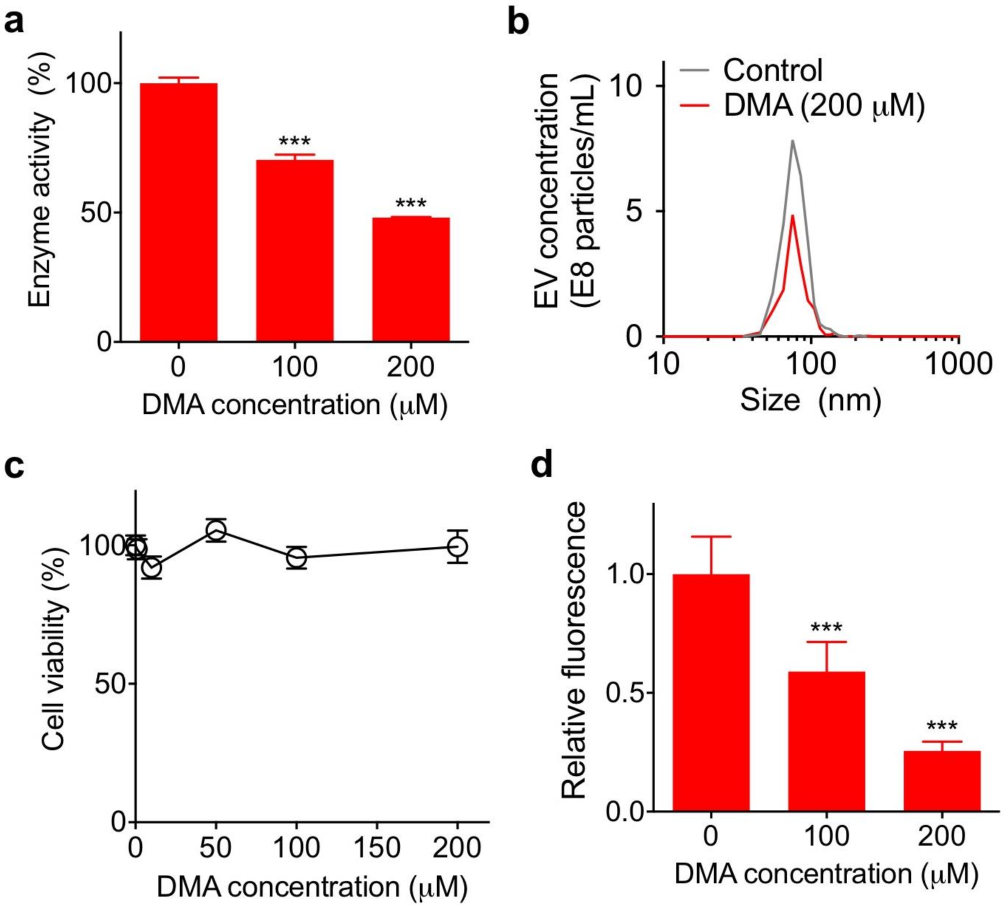

Supplementary Figure 5. Dimethyl amiloride (DMA) treatment for inhibition of exosome secretion. (a) Relative amount of exosomes produced from DMA-pretreated cells. HeLa cells were treated with 0, 100, and 200 μM DMA for 4 h. After 24 h, the secreted exosomes were collected and acetylcholinesterase activity was measured for exosome quantification. (b) Concentration of EVs produced from DMA-treated cells. HeLa cells were incubated for 4 h in the absence or presence of 200 μM DMA. After 24 h, the secreted EVs were collected, and the concentration was measured by nanoparticle tracking analysis. (c) Cell viability after DMA treatment. Cells were treated with 200 μM DMA for 4 h and further incubated for 24 h. MTT assay was performed for measuring cell viability. (d) Fluorescence quantification of EVs produced from fluorophore-FL-treated cells after DMA treatment. DMA-pretreated cells were treated with fluorophore-FLs for 1 h and further incubated for 24 h. The secreted EVs were collected using the ultracentrifugation protocol and their fluorescence was measured using a spectrofluorometer. Data are means ± s.e.m. [n = 3 for (a), n = 4 for (c) and (d), ***$p < 0.001$, one-way ANOVA with Tukey's post hoc test].

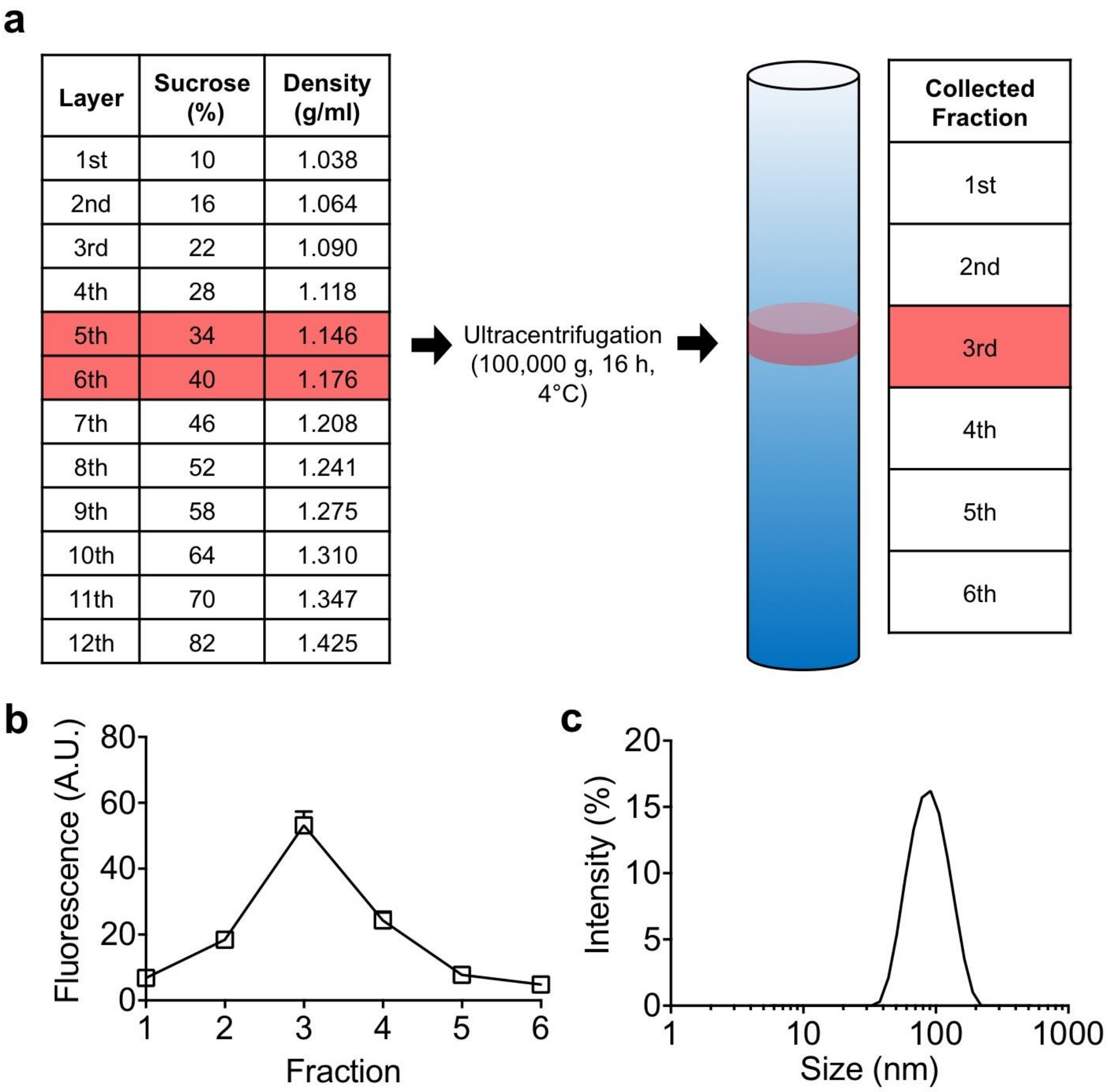

Supplementary Figure 6. EV purification using sucrose gradient ultracentrifugation. (a) Sucrose gradient and density table. After ultracentrifugation, EVs were enriched in the third fraction which consists of 5th and 6th layer with density of 1.146 and 1.176 g/ml, respectively, which was in agreement with the previous literature[1]. (b) Fluorescence intensity of each collected fraction. (c) Representative size distribution of EVs in the third fraction. Data are means ± s.e.m. (n = 3).

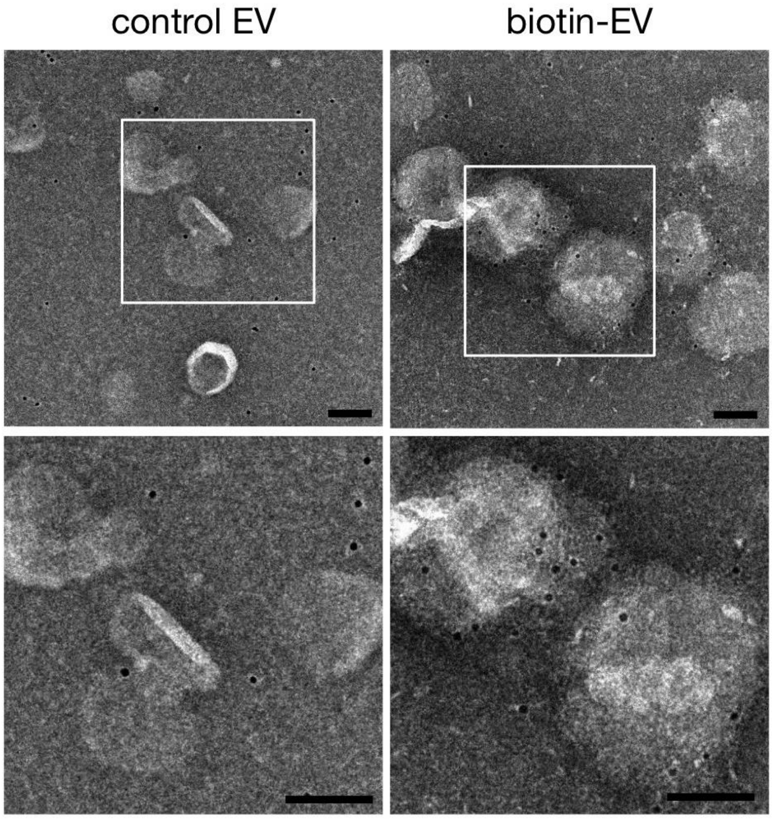

Supplementary Figure 7. Representative TEM images of unmodified EVs and biotin-EVs after streptavidin-nanogold staining. EVs from the cells treated with either PBS (control) or biotin-FLs were stained with streptavidin-nanogold(10 nm). EVs from biotin-FL-treated cells showed binding of gold nanoparticles on the surface while EVs from PBS-treated cells did not. Scale bars indicate 100 nm.

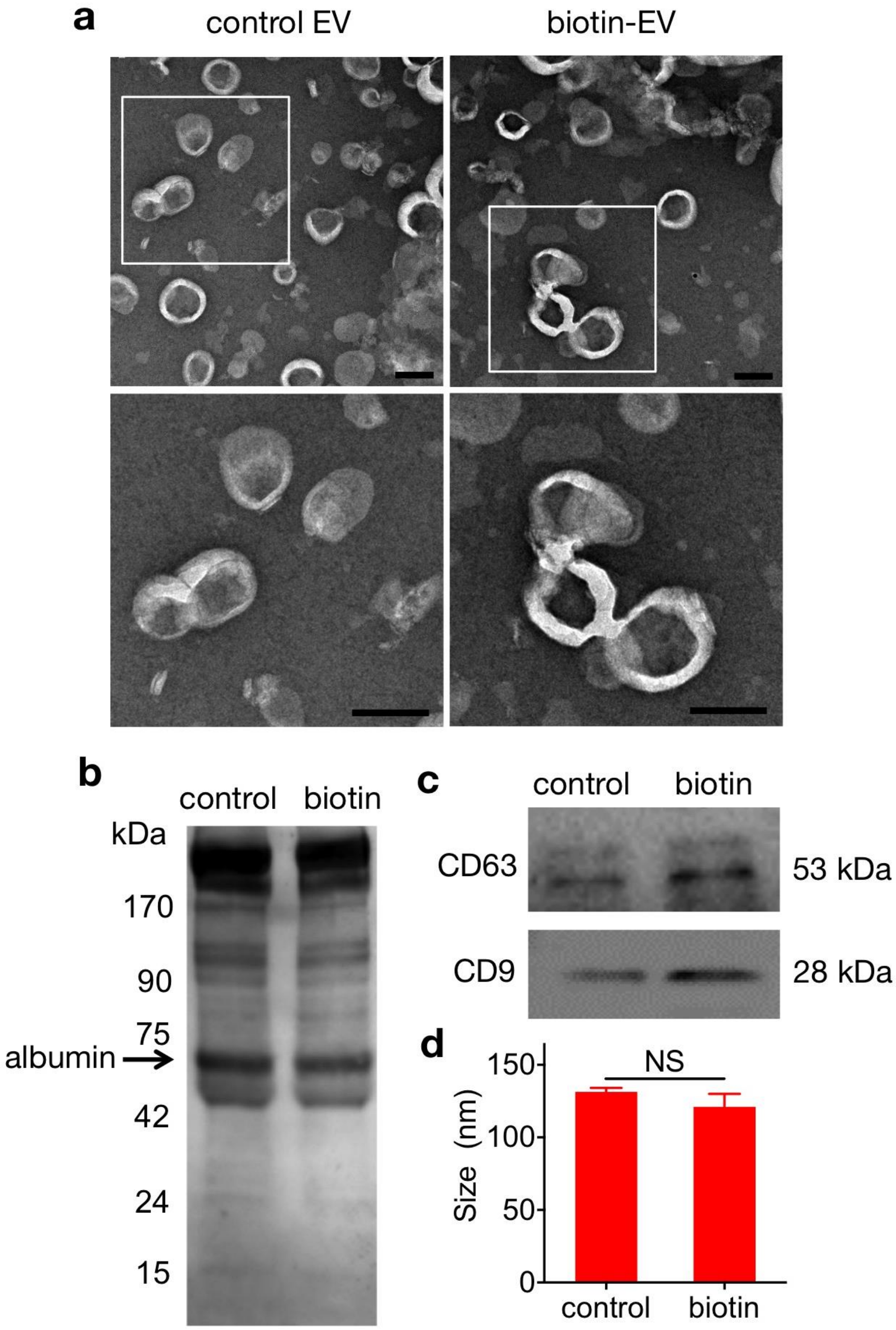

Supplementary Figure 8. Morphology, protein profile and size of EVs after biotin-FL treatment. (a) Representative TEM images of EVs from cells treated with either PBS (control) or biotin-FLs. Scale bars indicate 100 nm. (b) Coomassie blue staining of protein extracts from EVs from cells treated with either PBS (control) or biotin-FLs. (c) Western blot analysis of of exosomal marker proteins CD63 and CD9 on the EVs from cells treated with either PBS (control) or biotin-FLs. (d) Hydrodynamic size of EVs from cells treated with either PBS (control) or biotin-FLs. Data are means ± s.e.m. (n = 3; NS, not significant; Student's *t* test).

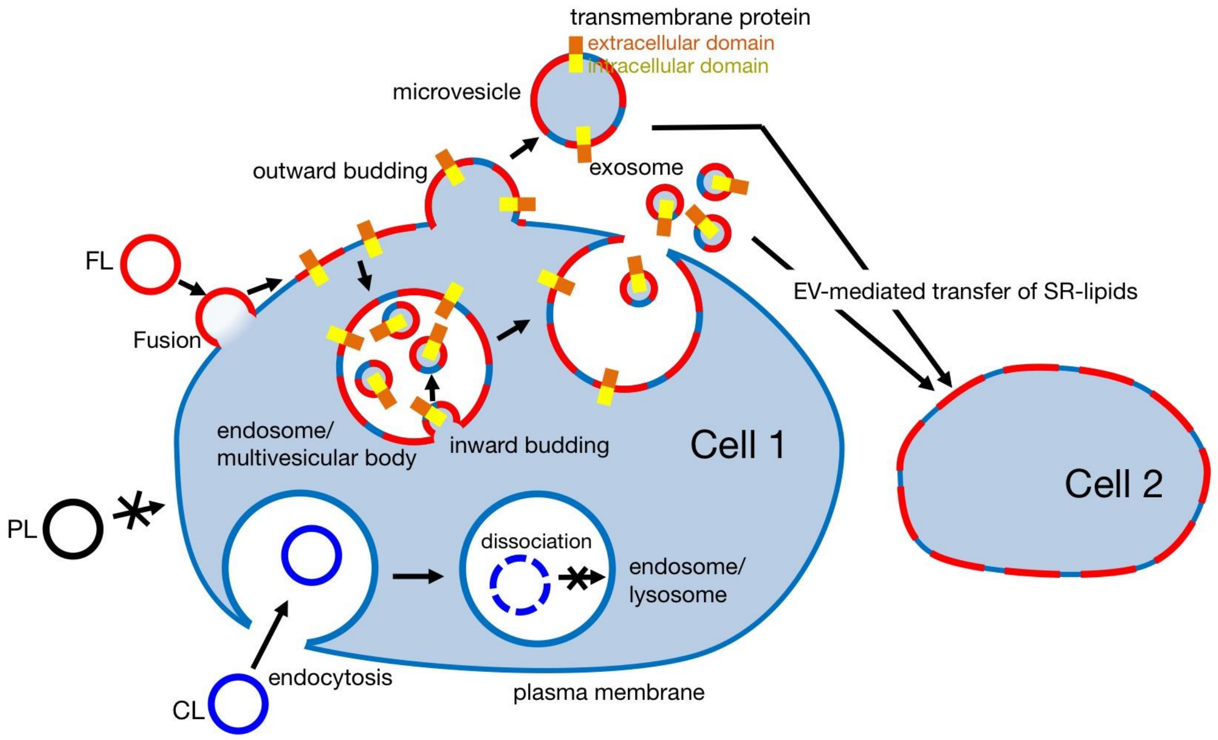

Supplementary Figure 9. Schematic showing mechanism of EV incorporation and EV-mediated intercellular transfer of SR-lipids for each type of liposomes. FLs transfer SR-lipids selectively into plasma membrane through membrane fusion, making them to become building blocks of plasma membrane structure. In one way, the SR-lipids can be incorporated into the microvesicle membrane because they are formed by outward budding of plasma membrane. In another way, the SR-lipids can be incorporated into exosome membrane by inward budding of endosomal membrane. CLs, however, go through rapid endocytosis and localize into endosomes/lysosomes where they are dissociated. This endocytosis hinders effective transfer of SR-lipids to cellular membrane, thereby lowering the efficacy of SR-lipid incorporation into EV membrane subsequently. In addition, PLs have less chance of SR-lipids to be incorporated into EVs secreted by the cells because they are poorly interacted with cells.

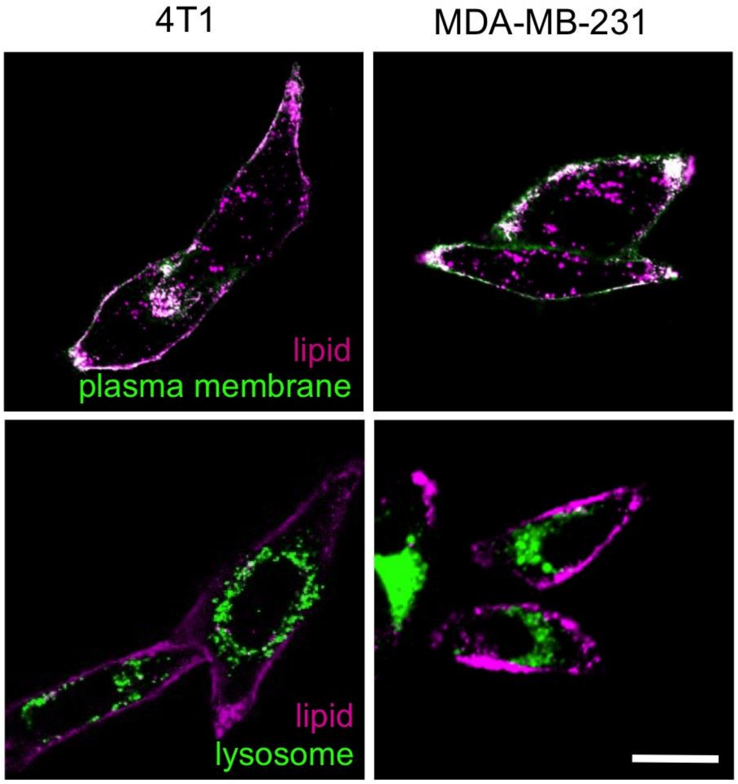

Supplementary Figure 10. Colocaliztaion of fluorophore-lipids with plasma membranes. Confocal fluorescent microscopic images of tumor cells (4T1 and MDA-MB-231) after FL-mediated delivery of fluorophore-lipids (magenta). Cells were treated with fluorophore-FLs for 1 h and then imaged. . The plasma membrane and lysosome were stained with CellMask and LysoTracker (green), respectively. Scale bar indicates 20 μm.

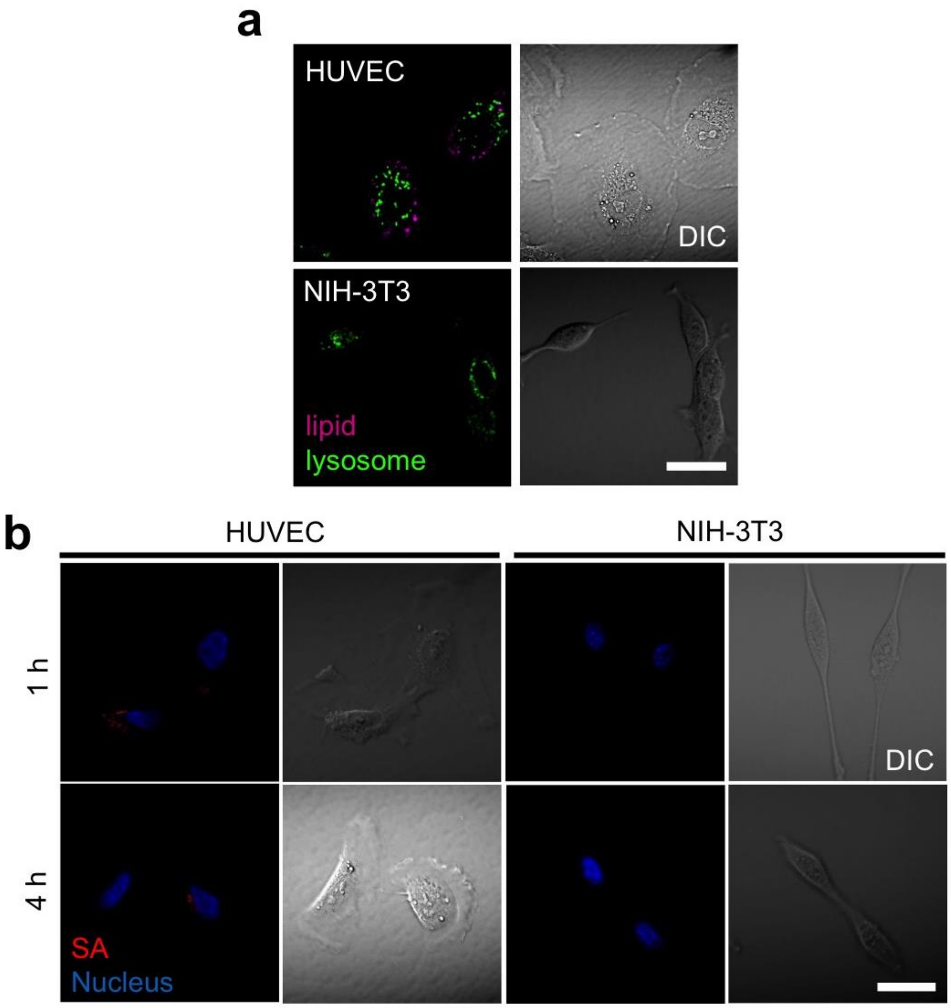

Supplementary Figure 11. FL treatment to endothelial and fibroblast cells. Human Umbilical Vein Endothelial Cells (HUVEC) and NIH-3T3 fibroblast cells were used as non-parenchymal cells. (a) Confocal fluorescent microscopic images of HUVEC and NIH-3T3 cells after FL-mediated delivery of fluorophore-lipids (magenta). Cells were treated with fluorophore-FLs for 1 h and then imaged. Lysosomes were stained with LysoTracker (green). (b) Confocal fluorescent microscopic images of HUVEC and NIH-3T3 cells treated with fluorophore-SA (red) 1 or 4 h after FL-mediated biotin-lipid delivery. Nuclei were stained with Hoechst (blue). Scale bar represents 20 μm.

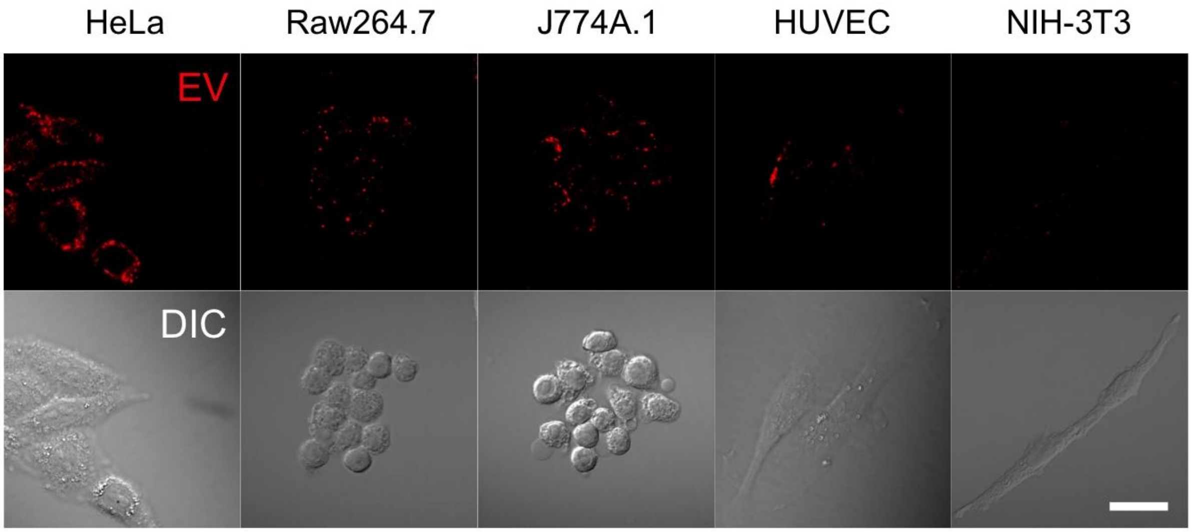

Supplementary Figure 12. Treatment of tumor cell-derived EVs to macrophages (raw264.7 and J774A.1), fibroblasts (NIH-3T3), tumor (HeLa) and endothelial cells (HUVEC). To generate tumor cell-derived EVs containing fluorophore-lipids, HeLa cells were treated with fluorophore-FLs for 4 h and the secreted EVs were isolated using ultracentrifugation. Each type of cells was then treated with HeLa-derived EVs containing fluorophore-lipids for 2 h, and imaged with confocal microscopy. Scale bar represents 20 μm.

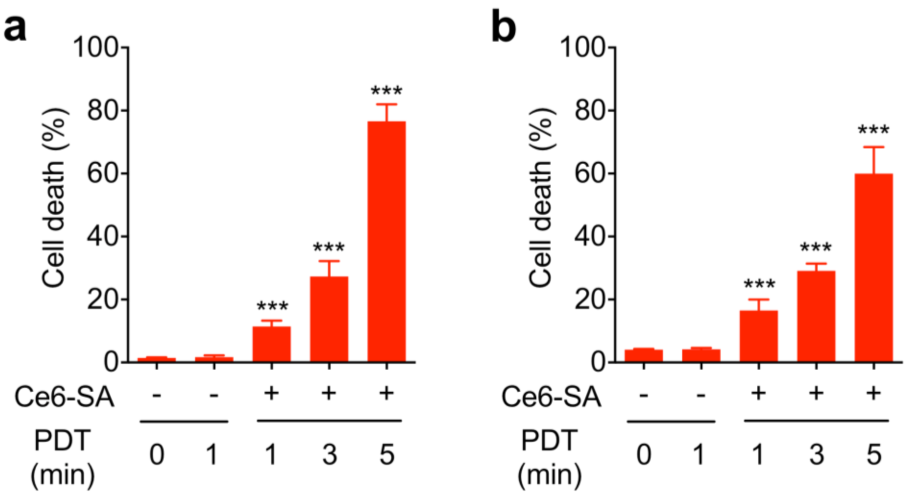

Supplementary Figure 13. Irradiation dose-dependent cell death after PDT. (a and b) 4T1 (a) or MDA-MB-231 (b) cells were treated with Ce6-SA for 1 h after FL-mediated biotin-lipid delivery, washed, and then irradiated for PDT. Data are means ± s.e.m. ($n = 3$, ***$p < 0.001$ compared to untreated controls using Student's *t* test).

a

	Biotin-FL	Ce6-FL
Zeta potential (mV)	15.1	- 4.5
Hydrodynamic size (nm)	126.6	128.1

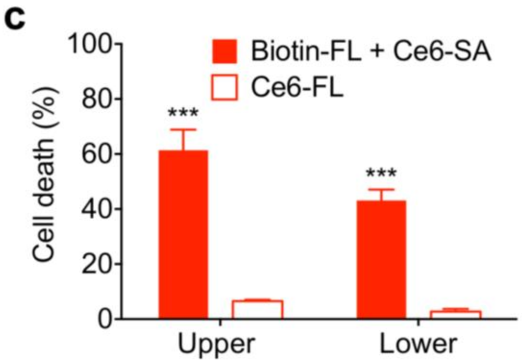

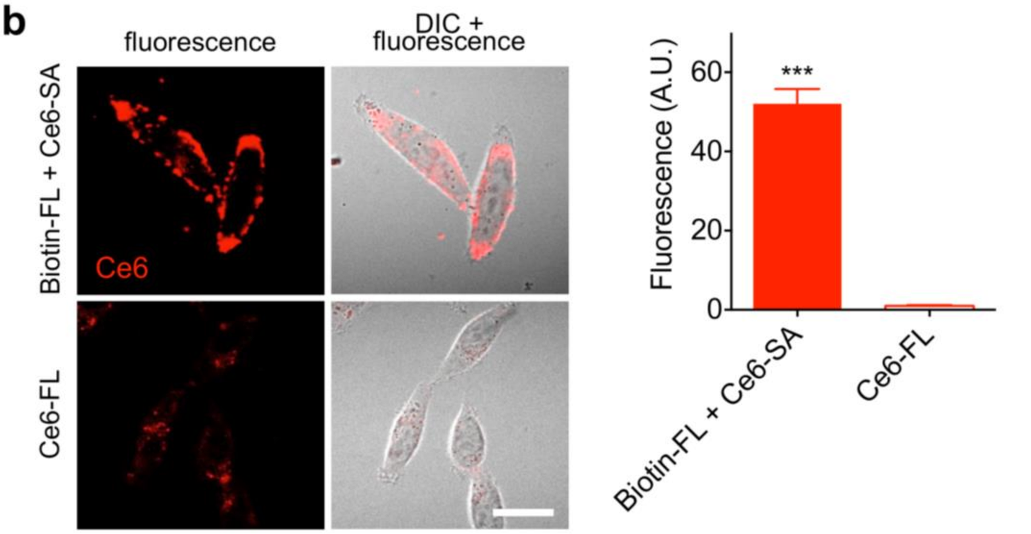

Supplementary Figure 14. Direct delivery of Ce6-lipids to plasma membrane using FLs. (a) Zeta potential and hydrodynamic size of biotin-FLs and Ce6-FLs. (b) Confocal fluorescent microscopic images and fluorescence quantification of HeLa cells treated with biotin-FLs followed by Ce6-SA or treated directly with Ce6-FLs. Data are means ± s.e.m. (n = 25, ***p < 0.001 compared to Ce6-FL using Student's *t* test). Scale bar is 20 μm. (c) Phototoxicity of HeLa cells in the upper transwell filter and the lower chamber either treated with Ce6-SA 4 h after treatment of cells in the upper filter with biotin-FLs or 4 h after direct treatment of cells in the upper filter with Ce6-FLs. Data are means ± s.e.m. (n = 5; ***p < 0.001 Student's *t* test).

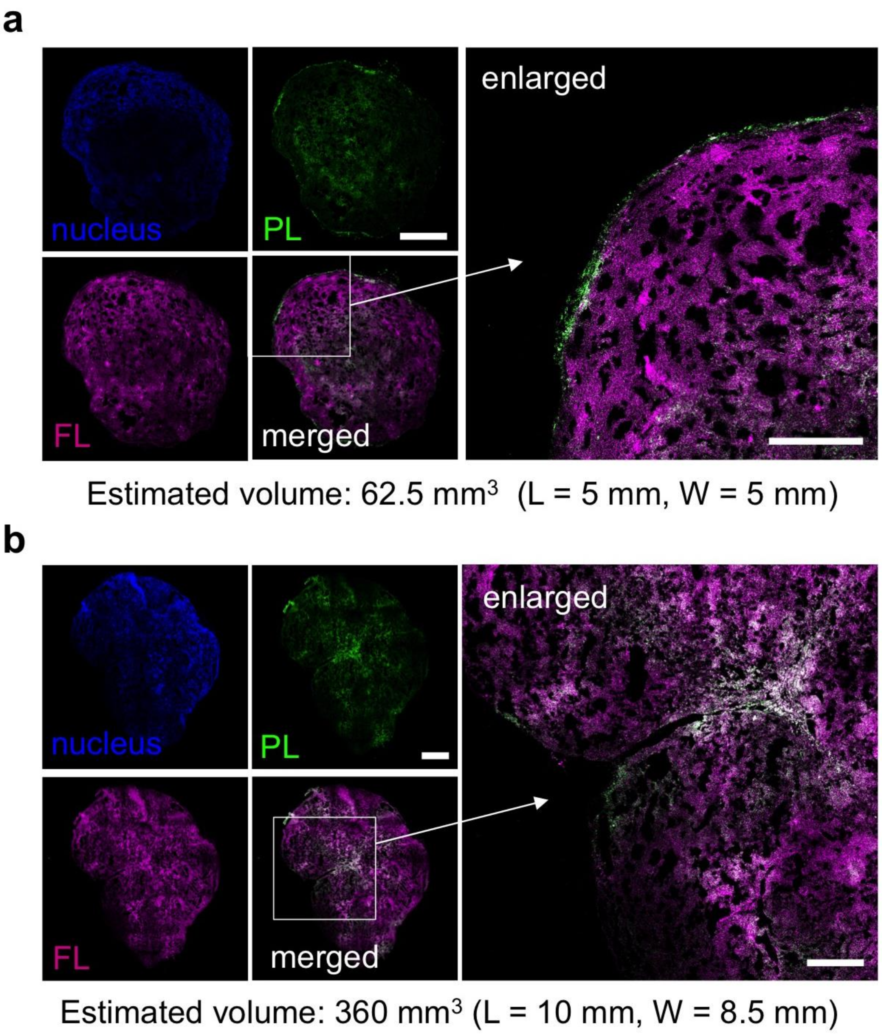

Supplementary Figure 15. Distribution of fluorophore-lipids delivered by FLs or PLs in 4T1 tumors with difference size. (a and b) Representative confocal microscopic images of tumor sections from the 4T1 tumors with estimated volumes of 62.5 mm^3 (a) and 360 mm^3 (b) collected at 24 h after intravenous co-injection of FLs (magenta) and PLs (green) incorporating fluorophore-lipids. Nuclei were stained with Hoechst (blue). Scale bars represent 1 mm.

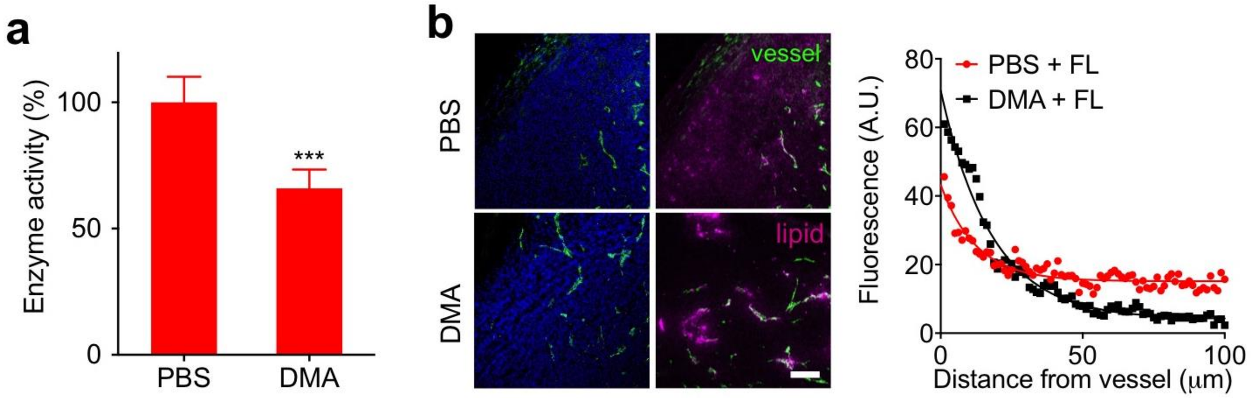

Supplementary Figure 16. Effect of systemic exosome depletion on tumor penetration of fluorophore-lipids. (a) Quantification of blood EVs after DMA treatment. Mice bearing 4T1 tumors were intraperitoneally injected with PBS or DMA daily for 3 days. Bloods were collected from the mice and EVs in the blood were quantified by measuring acetylcholinesterase activity. (b) Fluorescence images of PBS and DMA-treated 4T1 tumor sections and accumulation profiles of fluorophore-lipids after intravenous injection of fluorophore-FLs. PBS or DMA-treated mice were injected with FLs containing fluorophore-lipids (magenta). At 24 h, tumor samples were collected, and distribution of fluorophore-lipids was examined by confocal microscopy. Nuclei were stained with Hoechst (blue) and vessels with CD31 (green). Scale bar represents 100 μm. Data are means ± s.e.m. [n=4 , ***$p < 0.001$, Student's *t* test].

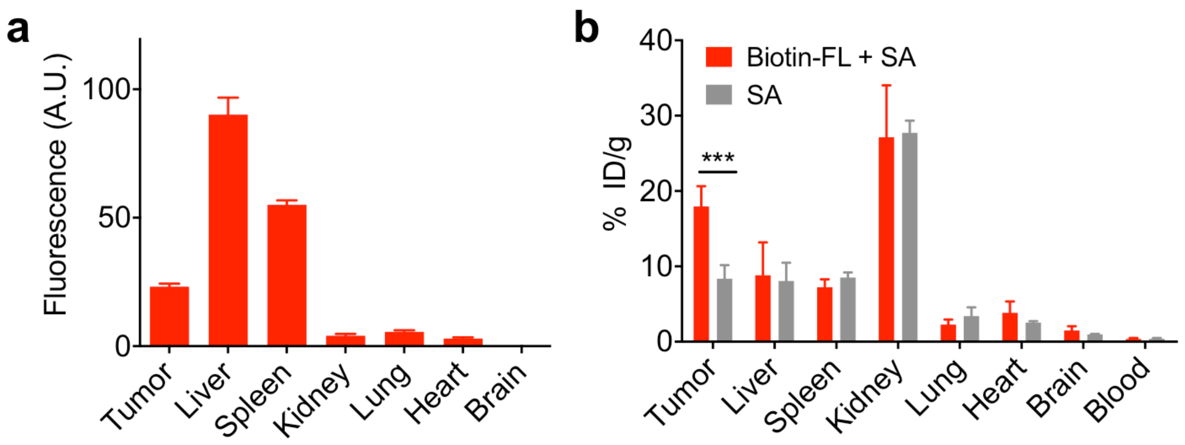

Supplementary Figure 17. Biodistribution of biotin-FL and SA. (a) Biodistribution of Cy7-conjugated-FLs (Cy7-FLs). Cy7-FLs were intravenously injected into mice bearing 4T1 tumors and their organs were harvested 1 day after liposome injection. Biodistribution of Cy7-FLs was visualized and quantified with *ex vivo* organs using NIR fluorescent imaging system. (b) Biodistribution of Alexa555-conjugated SAs (Alexa555-SA). Alexa555-SA was intravenously injected into mice bearing 4T1 tumors 1 day after biotin-FL or PBS injection, and their organs and blood were harvested 2 days after Alexa555-SA injection. Biodistribution of Alexa555-SA was quantified with fluorescence measurements of the homogenized organs and blood. Data are means ± s.e.m. (n = 4, ***$p<0.001$, Student's *t* test).

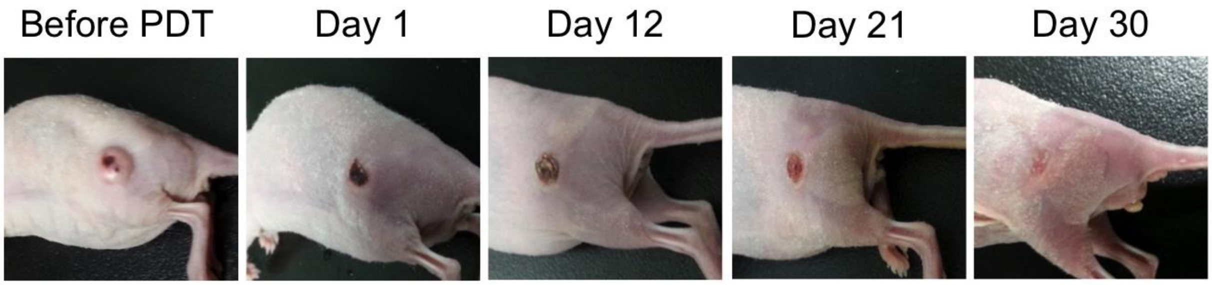

Supplementary Figure 18. Complete regression of a representative MDA-MB-231 tumor after cooperative membrane-targeted PDT.

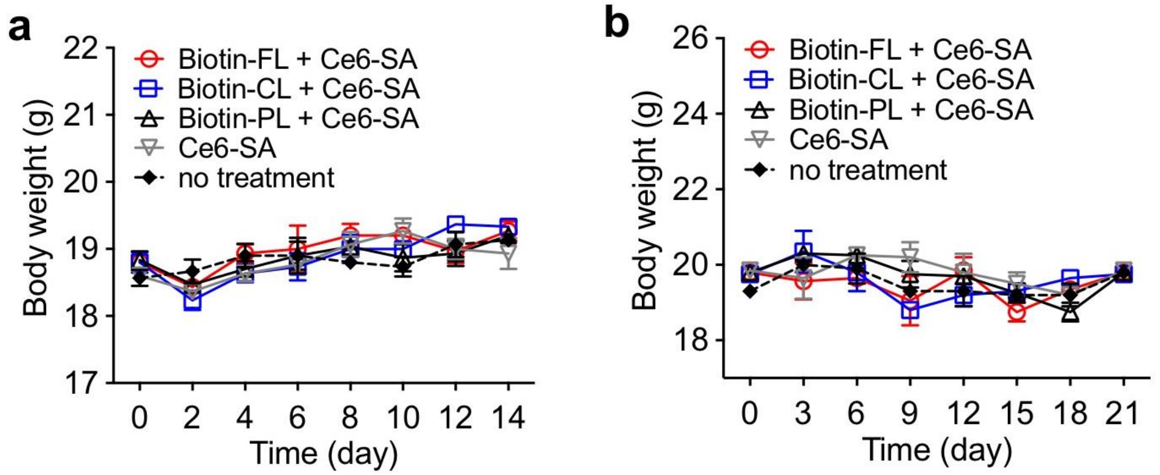

Supplementary Figure 19. Body weight change after PDT. (a and b) Body weight change were monitored after PDT in 4T1 (a) and MDA-MB-231 (b) tumors. Data are means ± s.e.m (n = 6).

Supplementary Methods

Heparin treatment

HeLa cells were treated with 140 μM FLs containing fluorophore-lipids for 1 h, washed thoroughly to remove free liposomes in the media, and further incubated for 24 h. The secreted EVs were isolated from the supernatant using the ultracentrifugation protocol and resuspended in PBS. Fresh HeLa cell were treated with 100 μg/ ml EVs containing fluorophore-lipids in the absence or presence of 100 μg/ ml heparin (sigma) for 2 h, and then imaged with confocal microscopy (Nikon, Tokyo, Japan).

Dimethyl amiloride treatment

For verification of EV depletion, HeLa cells were treated with 0, 100 or 200 μM dimethyl amiloride (DMA, inhibitor of exosome secretion[2], Abcam) for 4 h and incubated for 24 h before collection of supernatants. After EV isolation, the amount of secreted EVs was quantitated by measuring the activity of acetylcholinesterase (enzyme specifically present in the EV). Briefly, 25 μL of the EV fraction was suspended in 100 μL PBS and incubated with 1.25 mM acetylthiocholine (Sigma-Aldrich) and 0.1 mM 5.5'-dithiobis(2-nitrobenzoic acid) (Sigma-Aldrich) in a final volume of 1 mL. The solution was incubated at 37ºC for 20 min and the absorbance at 412 nm was measured. For quantification of EV concentration, the EVs produced from DMA-treated cells were collected using the ultracentrifugation protocol and diluted to obtain between 10 and 100 particles per image. Nanoparticle tracking analysis (NTA) measurements were performed to quantify the concentration of secreted EVs was using a

NanoSight NS300 (Malvern). To observe cell viability after DMA treatment, HeLa cells were treated with 200 μM DMA for 4 h and incubated for 24 h. MTT assay was performed for measuring cell viability. To observe EV incorporation of functional lipids, HeLa cells were incubated for 4 h in the absence or presence of 100 or 200 μM DMA. Then, the cells were treated with 140 μM FLs containing fluorophore-lipids for 1 h, washed thoroughly to remove free liposomes in the media, and further incubated for 24 h. The secreted EVs were collected using the ultracentrifugation protocol and their fluorescence was measured by using a spectrofluorometer (Gemini XPS; Molecular Devices).

Sucrose gradient ultracentrifugation

EV pellet was prepared from 200 ml of cell culture medium from fluorophore-FL-treated HeLa cells using the ultracentrifugation protocol and resuspended in 100 ul of PBS. EV pellet was resuspended in 100 ul of PBS. For EV purification, 10 - 90% (10, 16, 22, 28, 34, 40, 46, 52, 58, 64, 70, and 90%) sucrose stocks were prepared with PBS. The EV solution was mixed with 1 ml of 90% sucrose stock solution (final sucrose concentration = 82%) and transferred into 13.2 ml ultra-clear Beckman Ultracentrifuge tubes. Gradient was overlayed slowly on top of the EV solution starting with 1 ml of 70% sucrose solution (from the highest to the lowest sucrose concentration). The end of the pipette tip was in contact with the inside wall of the ultracentrifuge tube and the solution was slowly added into the tube. Ultracentrifugation was performed at 4°C (100,000g for 16 h). After ultracentrifugation, 2 ml fractions from the top to bottom were collected. Each fraction was examined for fluorescence and hydrodynamic size of EVs.

Transmission electron microscopy

The EVs produced from biotin-FL treated HeLa cells were fixed in 2% paraformaldehyde (Sigma-Aldrich) and stored at 4°C before use. 5 µl of resuspended EVs was deposited on Formvar-carbon coated EM grids (Ted Pella, Inc.) for 20 min until it dries. For nanogold-staining, the grids were blocked using blocking solution (1% BSA, 5% goat serum, and 0.02% Tween) for 10 min, and incubated with streptavidin-nanogold (10 nm, 1/50 diluted, Sigma-Aldrich) for 30 min. The grids were then washed with PBS for 6 times. For stabilization, the grids were transferred to a 50 µL drop of 1% glutaraldehyde for 5 min before transferring to a 100 µL drop of distilled water for 2 min. This was repeated 7 times for a total of 8 water washes. The grids were kept wet on the side of the membrane during all steps, but dry on the opposite side. For negative staining, the grids were placed onto a 50 µl drop of 2 % phosphotungstic acid (Sigma-Aldrich) for 2 min. Transmission electron microscopic images were obtained using a JEM-2100F HRTEM operating at 200 kV (JEOL).

Size measurement

The EVs produced from fluorophore/biotin-FL-treated cells were isolated using the ultracentrifugation protocol and resuspended in PBS. The hydrodynamic size of EVs was measured using dynamic light scattering (Zetasizer Nano ZS90; Malvern Instruments, Malvern, UK).

Western blot

The EVs produced from biotin-FL-treated cells were isolated using the ultracentrifugation protocol and resuspended in PBS. The protein concentration of EVs was measured by performing BCA assay. The EVs were washed and concentrated using a 100K Amicon centrifugal filter (Millipore, Billerica, MA, United States) to obtain 2 mg/ml protein concentration. 30 μl of EV solution was mixed with 10 μl of 4x Laemmli buffer (Bio-Rad) and boiled for 10 min at 95°C. Proteins were resolved by SDS-PAGE, transferred to polyvinylidene fluoride (PVDF) membrane. The protein profile of EVs was visualized on the PVDF membrane using Coomassie blue staining according to the manufacture's protocol (Bio-rad). For immunoblotting, the PVDF membrane was blocked in 5% skim milk for 1 h. The membranes were treated with CD63 antibody (Santa Cruz Biotechnology, Catalog number sc-15363) or CD9 antibody (Santa Cruz Biotechnology, Catalog number sc-9148), and incubated overnight at 4°C. The membranes were washed 3 times with Tris-buffered saline with 0.1% Tween 20 (TBST) and treated with Horseradish Peroxidase-linked secondary antibody (Santa Cruz Biotechnology, Catalog number sc-2030) for 1 h. The membranes were washed 3 times with TBST. Protein bands were detected using X-ray film and enhanced using chemiluminescence reagent (Bio-Rad, Catalog number 1705061).

Cell treatment with tumor cell derived EVs

To prepare tumor cell-derived EVs containing fluorophore-lipids, HeLa cells were treated with 140 μM FLs containing fluorophore-lipids for 4 h, washed thoroughly to remove free liposomes in the media, and further incubated for 24 h. The secreted EVs were isolated from the

supernatant using the ultracentrifugation protocol and resuspended in PBS. Tumor cells (HeLa), macrophages (Raw264.7 and J774A.1) or non-parenchymal cells (HUVEC and NIH-3T3) were treated with tumor cell-derived EVs containing fluorophore-lipids for 2 h and imaged with confocal microscopy (Nikon, Tokyo, Japan).

In vivo fluorescence tissue imaging

To observe SR-lipid delivery in small and large tumors, tumor models were generated by implanting 5×10^5 4T1 cells in 5-week-old female Balb/c mice. When the tumor volume reached approximately 60 or 360 mm^3, mice were co-injected with 200 μl of 5 μM FLs and PLs containing fluorescent lipids. After 24 h, the mice were sacrificed for histological analysis. The tumor sections were then examined under a confocal microscope (Nikon).

Biodistribution

For biodistribution of SR-lipids, Cy7-FLs were intravenously injected into mice bearing 4T1 tumors and their organs were harvested 1 day after liposome injection. Biodistribution of Cy7-FLs was visualized and quantified with *ex vivo* organs using NIR fluorescent imaging system (Odyssey, LI-COR Biosciences). For biodistribution of targeting agents, Alexa Fluor 594-conjugated SA was intravenously injected into mice bearing 4T1 tumors 1 day after biotin-FL or PBS injection, and their organs and blood were harvested 2 days after SA injection. Biodistribution of Alexa Fluor 594-conjugated SA was quantified with fluorescence

measurements of the homogenized organs and blood. All experiments in this section were repeated at least three times and they showed similar results.

Supplementary References

1 Witwer, K. W. *et al.* Standardization of sample collection, isolation and analysis methods in extracellular vesicle research. *Journal of Extracellular Vesicles* **2**, 10.3402/jev.v3402i3400.20360, doi:10.3402/jev.v2i0.20360 (2013).

2 Zhang, L. *et al.* Microenvironment-induced PTEN loss by exosomal microRNA primes brain metastasis outgrowth. *Nature* **527**, 100-104, doi:10.1038/nature15376 (2015).
